# Resistance of aerobic granular sludge microbiomes to periodic loss of biomass

**DOI:** 10.1016/j.bioflm.2023.100145

**Published:** 2023-07-28

**Authors:** Raquel Liébana, Oskar Modin, Frank Persson, Malte Hermansson, Britt-Marie Wilén

**Affiliations:** a Division of Water Environment Technology, Department of Architecture and Civil Engineering, Chalmers University of Technology, Sven Hultins gata 6, SE 412 96, Gothenburg, Sweden; b AZTI, Marine Research Division, Basque Research Technology Alliance (BRTA), Txatxarramendi Ugartea z/g, 48395, Sukarrieta, Bizkaia, Spain; c Department of Chemistry and Molecular Biology, University of Gothenburg, Medicinaregatan 9E, SE-413 90, Gothenburg, Sweden

**Keywords:** Granular sludge, Disturbance, Microbial succession, Null model, Resilience

## Abstract

Granular sludge is a biofilm process used for wastewater treatment which is currently being implemented worldwide. It is important to understand how disturbances affect the microbial community and performance of reactors. Here, two acetate-fed replicate reactors were inoculated with acclimatized sludge and the reactor performance, and the granular sludge microbial community succession were studied for 149 days. During this time, the microbial community was challenged by periodically removing half of the reactor biomass, subsequently increasing the food-to-microorganism (F/M) ratio. Diversity analysis together with null models show that overall, the microbial communities were resistant to the disturbances, observing some minor effects on polyphosphate-accumulating and denitrifying microbial communities and their associated reactor functions. Community turnover was driven by drift and random granule loss, and stochasticity was the governing ecological process for community assembly. These results evidence the aerobic granular sludge process as a robust system for wastewater treatment.

## Highlights

• Disturbances did not generally affect the performance of granular sludge reactors.
• The microbial community was overall resistant to disturbances.
• Community variability was mainly driven by changes in alpha-diversity.
• Community turnover was driven by stochasticity.

## 1 Introduction

Wastewater treatment using biofilm processes is becoming increasingly common. This is due to the existence of different habitats within the biofilm which favors the coexistence of different microbial groups, allowing the removal of organic matter and nutrients in the same reactor [1]. Also, biofilms have long retention times and slow growing bacteria (for instance those involved in nitrogen and phosphorus removal) can be kept in the reactors by growing in a biofilm. Granular sludge is a biofilm process that has gained interest both for aerobic and anaerobic wastewater treatment [2,3]. Currently, the aerobic granular sludge technology has been implemented in more than 80 full-scale wastewater treatment plants worldwide [4]. Sequencing batch reactors (SBRs) are used for the sludge granulation, where several conditions (such as high up-flow air velocity, feast-famine operation, and short settling time) are applied to force bacteria to switch from a planktonic to an aggregate growth mode and to select for suspended biofilms with spherical shape and excellent settleability [[5], [6], [7], [8]]. Since different ecological niches are created by the substrate gradients within the granules, they have the ability to remove carbon, nitrogen and phosphorous simultaneously [8,9]. Additionally, the biofilm growth mode allows for a high reactor biomass concentration, rendering increased process rates. Altogether, it makes this technology a compact and less energy demanding process for wastewater treatment. For comparison, the aerobic granular process has been reported to be 50% and 13% more compact than the activated sludge process with biological phosphorous removal and the fixed-film activated sludge (IFAS) process, respectively, displaying also a lower estimated energy demand (23% lower than activated sludge process, 35% lower than IFAS, or 50–70% lower than membrane bioreactors) [10].

The formation of granules and the general operation to achieve removal of the desired pollutants from wastewater can, to some extent, be controlled by the engineer. However, a major concern in engineered ecosystems is the maintenance of stable process performance [11] despite operational disturbances that frequently occur, and factors that are difficult to control, such as the influent composition (concentration of different substrates, presence of toxic pollutants, etc.) or the temperature [12]. In this regard, even when granular sludge is increasingly applied for wastewater treatment, there is still a need to broaden the knowledge on the effect of disturbances on the microbial community structure and function.

Wastewater treatment reactors often experience disturbances which could lead to a failure of the system, causing a poor reactor performance and the associated environmental and economical negative impact [13]. Disturbances are changes in environmental conditions or loss of biomass that could affect the microbial community structure and function [14,15]. These community changes could result from the influence of deterministic processes (e.g. selection), that would make community changes after a disturbance predictable and repeatable which is highly desired from an engineering perspective, and/or stochastic process (e.g. ecological drift, birth or death) that would lead to variable responses [[16], [17], [18]]. The extent of changes following a disturbance is determined by the ecosystem stability which depends on the ability of an ecosystem to remain unchanged after a disturbance (resistance) and the rate at which the community returns to a pre-disturbance state (resilience) [14]. Under disturbed conditions, a non-resistant microbial community may adapt to the newly created environment which will involve compositional changes. Previous studies have shown that these community changes not necessarily affect the performance in wastewater treatment bioreactors [[19], [20], [21], [22], [23]]. This is due to a high degree of functional redundancy often observed in microbial communities [24]. [23] observed an overall stable performance of aerobic granular sludge reactors when multiple perturbations of different nature (i.e., change in the food-to-microorganism (F/M), pH, lack of micronutrients) occurred in the reactors, regardless of the wastewater used (simple or complex) and despite the observed changes in the bacterial community composition. The authors identified multiple stable states where the functional stability was preserved, likely due to the presence of sufficient microbial functional redundancy. This is in line with the results obtained by Ref. [25]; who reported a stable reactor performance and functional recovery after perturbations of a pilot-scale anaerobic digester treating industrial wastewater due to the functional redundancy of the sludge community. However, even if a stable reactor performance has been observed under high microbial community dynamics, the opposite has also been observed, especially for complex functions [[26], [27], [28]].

In wastewater treatment bioreactors, sludge loss due to process malfunctioning is a disturbance that potentially could affect microbial community structure and function. Sudden changes in wastewater properties like those occurring due to heavy rain events or increased melt water-streams, which cause peak inflows into wastewater treatment plants, should be considered as process malfunctioning occur due to sludge loss. Indeed, granule biofilm development and granular stability has been reported to be affected by events involving reactor biomass loss [29]. Biomass washout events in granular sludge reactors have been reported associated to changes in wastewater properties, such as salinity [30,31] or temperature [[32], [33], [34]]. Additionally, sludge loss increases the F/M ratio, which is a factor that has a great importance in granular sludge systems as it is related to the granule stability, microbial diversity and the removal of the contaminants [[35], [36], [37], [38], [39]].

While full-scale wastewater treatment plants harbour complex ecosystems where replicated and controlled conditions are not achievable, laboratory-scale bioreactors offer the opportunity to study the microbial community dynamics during disturbances. In this study, we have challenged the microbiome of granular sludge biofilms by periodically removing half of the reactor biomass to study the impact of disturbances on the microbial community structure and the performance in granular sludge reactors. For this, two acetate fed replicate reactors were run in parallel for 149 days when disturbances were applied. Measurements of the compositional differences or turnover within (alpha-diversity) and between (beta-diversity) the reactor microbial community assemblages, together with null models, were used to study the ecological processes behind the community dynamics during the applied disturbances. We aimed to 1) examine whether the microbial community remained stable or changed under a disturbed regime, 2) assess if the functionality of the reactor was ensured and 3) assess the ecological processes governing the community assembly under periodical disturbances.

## 2 Experimental procedures

### 2.1 Experimental design

**Fig. 1:**
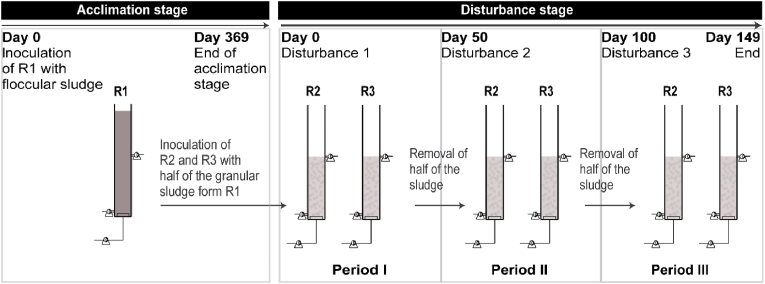
Schematic illustrating the experimental design. The different stages of the experiment and the disturbance timeline are shown. R2 was inoculated with half of the R1 sludge and R3 with the other half.

Three identical SBRs (R1, R2 and R3) were used in the experiment, which was divided into two stages: (1) acclimation and (2) disturbance (see Fig. 1). In the acclimation stage, reactor R1 was inoculated with activated sludge from the Hammargården wastewater treatment plant designed for biological nitrogen and phosphorus removal (Kungsbacka, Sweden) and operated at a settling time of 30 min for 369 days. In the disturbance stage, each of the reactors R2 and R3 were inoculated with half of the sludge from reactor R1, and operated identically for 149 days. During this disturbance stage, the settling time was set to 2 min and three disturbances were performed, defining three periods accordingly (see Fig. 1). The disturbances were applied by removing half of the volume of the settled biomass in the reactors, which resulted in an instantaneous doubling of F/M ratio.

### 2.2 Set-up and operation of reactors

The SBRs, previously described in detail [40], had working volumes of 3 L. Synthetic wastewater was used and consisted of 994.2 mg/L NaCH_3_COO, 443.8 mg/L NH_4_Cl, 139.5 mg/L K_2_HPO_4_, 56.5 mg/L KH_2_PO_4_, 12.5 mg/L MgSO_4_·7H_2_O, 15.0 mg/L CaCl_2_, 10.0 mg/L FeSO_4_·7H_2_O, and 1 mL/L micronutrient solution (as described in Ref. [40]). The feed flow resulted in an organic loading rate of 2 kg COD/m^3^d^1^, *N*-load of 0.3 kg NH_4_–N/m^3^d^1^ and P-load of 0.1 kg PO_4_–P/m^3^d^1^ resulting in a COD:N:P ratio of 20:3:1. The reactors were operated at room temperature (20–22 °C) with a volumetric exchange ratio of 43%. A 4-h cycle was used consisting in 5 min filling, 55 min anaerobic, 143 min aerobic, 30 min settling, 5 min withdrawal and 2 min idle in the acclimation stage and 5 min filling, 55 min anaerobic, 173 min aerobic, 2 min settling, 5 min withdrawal and 2 min idle in the disturbance stage.

### 2.3 Analytical methods

Concentrations in the effluent of total organic carbon (TOC) and total nitrogen (TN) were measured with a TOC-TN analyser (TOC-V, Shimadzu, Japan), and acetate, ammonium, nitrite, nitrate and phosphorus were measured using a Dionex ICS-900 ion chromatograph. The samples were immediately centrifuged, filtered (0.45 μm) and stored at −20 °C until analysis. Total suspended solids and volatile suspended solids in the in the reactor and in the effluent, and sludge volume index (SVI) were measured according to standard methods [41]. Microscopy was performed using an Olympus BX60 light microscope (Olympus Sverige AB, Solna, Sweden) with particle size assessment by measuring the diameter of 15 random granules using the ImageJ software [42]. Cycle studies were performed on day 100, when disturbance 3 was exerted to the reactors. To avoid biases while sampling the reactors, a flexible plastic tube (ø 1 cm) attached to a syringe was used to sample the reactor at different heights during the aerobic phase and in the upper third of the sludge bed during the anoxic phase.

### 2.4 DNA extraction, amplification and sequencing

During the disturbance stage, a total of 54 samples were collected for DNA analysis, distributed in 27 sample points for reactor R2 and R3. The 16S rDNA library was constructed as described in Ref. [40]. Shortly, the V4 region of the 16S rRNA gene was amplified using the forward primer 515′F (5′-GTGBCAGCMGCCGCGGTAA-3′) and the reverse primer 806R (5′-GGACTACHVGGGTWTCTAAT-3′), indexed according to Kozich et al. (2013). The PCR products were sequenced with a MiSeq using reagent kit v3 (Illumina). Additionally, six DNA extractions of the seed sludge (R1) and of the last day of operation of R2 and R3 were used as replicates.

### 2.5 Sequence processing and data analysis

Raw sequence reads were processed following the UNOISE pipeline [43]; [44]. with USEARCH v.11 [45]. Briefly, paired-end reads were merged with a maximum number of mismatches of 15 (5% of the read length) and a minimum of 80% of identity in the alignment. Merged reads were filtered (maximum expected error per read of 0.5), dereplicated (default settings) and denoised (minimum abundance of 4). The obtained amplicon sequencing variants (ASVs) were taxonomically classified with the SINTAX algorithm [46] based on the MiDAS database v.2.1 [47] and analysed in R version 3.4.1 (http://www.r-project.org). The dataset was rarefied, subsampling each sample to 70492 sequences. Basic R functions were used to perform Wilcoxon signed-rank tests. Heatmaps were created using the R package ampvis2 [48]. The sequences were aligned with the R package DECIPHER [49] and a maximum likelihood tree was generated with the FastTree 2 software [50] using the GTR + CAT (General Time Reversible with per-site rate CATegories) model of approximation for site rate variation and computation of Gamma20-based likelihood. Analysis of pairwise multivariate permutational analysis of variances (PERMANOVA) was conducted using adonis from the package pairwiseAdonis [51]. Time-decay rates were calculated as in Ref. [52]. Dissimilarities between samples were converted to similarities by subtracting from one. The time-decay rate was the linear slope of the log-transformed similarities plotted against the time difference between samples.

For each sample, alpha-diversity was calculated using Hill numbers [53]. The parameter q is the diversity order. At a q of 0, all ASVs are considered equally important and, at q of 1, the ASVs are given weight according to their relative abundance and for higher values of q, more weight is put on abundant ASVs. Hence, ^0^TD is the sample richness (i.e. the number of ASVs in a sample), ^1^TD is equivalent to the number of “common” ASVs and ^2^TD is equivalent to the number of “abundant” ASVs. For all pairwise comparisons of samples, beta-diversity was obtained using the same calculation framework. The beta-diversities were converted into dissimilarity indices constrained between 0 (two identical samples) and 1 (two samples with no shared ASVs) [54,55]. We also calculated diversity numbers which take the sequence dissimilarity into account using the framework described by Ref. [55]. The metric, here referred to as ^q^PD (phylogenetic diversity), represents the total effective phylogenetic distance between all ASVs in a sample. The Hill-based alpha-diversities and dissimilarities were calculated using qdiv [56]. Raw sequences were deposited in the NCBI Sequence Read Archive (SRA), BioProjectID PRJNA893179.

### 2.6 Null model analysis

Null models were used to analyse the taxonomic- and phylogenetic successional turnover between consecutive samples and between reactors. Taxonomic turnover was estimated with Raup-Crick-based measures using Hill-based dissimilarities (^q^RC), calculated within qdiv [56]. Briefly, a null distribution (999 randomizations) of pairwise dissimilarities was created. From the null distribution, a mean and a standard deviation of the expected dissimilarity between pairs of communities were calculated. The ^q^RC index was expressed as the difference in standard deviation units between the observed dissimilarity and the mean of the null distribution. Values > |2| were considered statistically significant. Values < −2 indicate that the two communities share more taxa than expected by chance whereas a value > 2 indicate that they share less taxa. Values < |2| indicates that the taxonomic turnover between the community pair was not different from the null expectation and that they were, therefore, influenced by stochastic factors [57].

The R package PICANTE [58] was used to calculate the β nearest-taxon index (βNTI), which measures if the phylogenetic turnover is greater or less than the null expectation. For this, the β mean nearest-taxon distance (βMNTD), which measures the mean phylogenetic distance between the most closely related ASVs in two communities, was first calculated based on relative abundance data, as in Ref. [59]. In each iteration (999 randomizations) the ASVs were moved randomly across the tips of the regional pool phylogeny and the resulting phylogenetic relationships were used to calculate the βMNTD_null_. The βNTI was calculated as the difference in standard deviation units between the observed βMNTD and the mean of βMNTD_null_ in the pairwise sample comparisons. A negative βNTI value means that the samples are more phylogenetically close to each other than expected by chance and a positive value means that the two samples are more phylogenetically distant from each other. Previous studies have found that short phylogenetic distances between ASVs, quantified using βMNTD and βNTI, reflect close ecological distances [[59], [60], [61]]. Pairwise comparisons with βNTI > |2| were considered statistically significant. Values < |2| were not significantly different from the null expectation, which indicate that stochastic factors influenced the phylogenetic turnover [57].

## 3 Results

### 3.1 The replicate reactors had similar performance

**Fig. 2:**
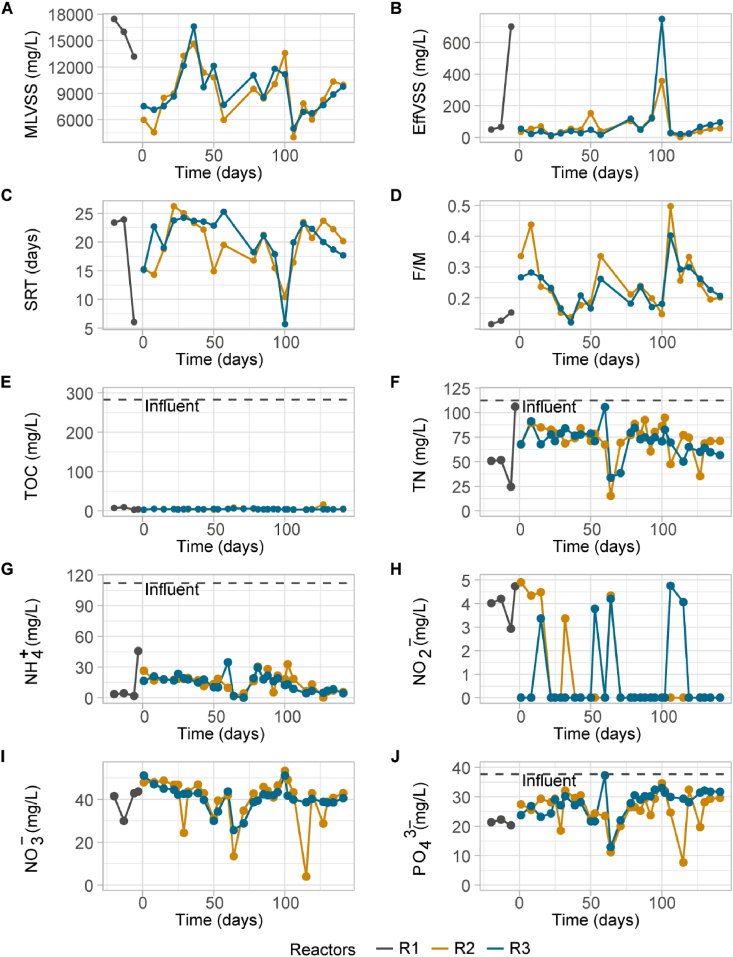
Sludge properties and process performance over time. **A**, reactor volatile suspended solids (MLVSS) concentrations; **B**, effluent volatile suspended solids concentrations (EffVSS); **C**, sludge retention time (SRT); **D**, food-to-microbe (F/M, mgCOD/mgMLVSS· d) ratio; **E**, effluent total organic carbon (TOC); **F**, effluent total nitrogen; **G**, effluent ammonium concentration (NH_4_^+^); **H**, effluent nitrite concentration (NO_2_^−^); **I**, effluent nitrate concentration (NO_3_^−^); **H**, effluent phosphate concentration (PO_2_^3−^); Horizontal dashed lines indicate the influent concentration.

The sludge in R1 was fully granulated after 116 days, displaying the granules an average diameter of 1.6 mm (SD = 0.8) during the remaining 253 days left of the acclimation stage. The acclimatized granules from R1 after 369 days of operation were used as inoculum for reactors R2 and R3 and had a mean diameter of 1 mm (SD = 0.3) and increased in size during the disturbance stage of the experiment (Figs. S1 and S2), displaying an average diameter of 2.3 mm (SD = 0.7) in R2 and 2.6 mm (SD = 0.7) in R3 at the end of the experiment. No effect caused by the disturbances was observed in terms of granule size increase (Fig. S1) and granule structure (Fig. S2). Furthermore, no statistical differences in granule size were found between reactors (Wilcoxon signed-rank p > 0.05, Table S1). The sludge parameters in R2 and R3 overall showed similar trends (Fig. 2A–C). During the disturbances, when half of the sludge was removed from the reactors, there was a decrease in suspended solids concentration in the reactors. The first disturbance reduced MLVSS from 16 g/L in the seed sludge to 13 and 7 in R2 and R3 respectively (day 0), while the second disturbance (day 50) caused a reduction from 11 g/L to 6 g/L in R2 and 12 g/L to 8 g/L in R3, and the third disturbance (day 100) caused a higher reduction in MLVSS, from 14 g/L to 4 g/L in R2 and 11 g/L to 5 g/L in R3. Consequently, the solids retention time (SRT) decreased (Fig. 2C), especially after the second disturbance in R2 (from 22 days to 15) and after the third disturbance in R3 (from 18 days to 6). The F/M ratio in turn increased (Fig. 2D), especially after the third disturbance in both reactors (from 0.15 to 0.5 in R2 and from 0.18 to 0.4 in R3). The SVI remained stable at 20 mL/g (SD = 4.8) and 17.5 mL/g (SD = 3.1) in R2 and R3, respectively during disturbance period I and increased to 23.9 mL/g (SD = 2.2) and 19.8 mL/g (SD = 2.8) in R2 and R3, respectively, during disturbance period II and to 27.4 mL/g (SD = 5.3) and 26.6 mL/g (SD = 3.6) in R2 and R3, respectively, during disturbance period III. After the disturbances, the concentration of suspended solids in the effluent increased, especially marked in the third disturbance (127 mg L^−1^ to 357 mg L^−1^ in R2 and 120 mg L^−1^ to 747 mg L^−1^ in R3). Hence, disturbance 3 resulted in a higher loss of biomass, thus changes in sludge parameters were most clearly observed and especially for R3. Statistical differences between reactors for volatile suspended solids in the effluent were observed in disturbance periods I and III (Wilcoxon signed-rank p < 0.05, Table S2).

**Fig. 3:**
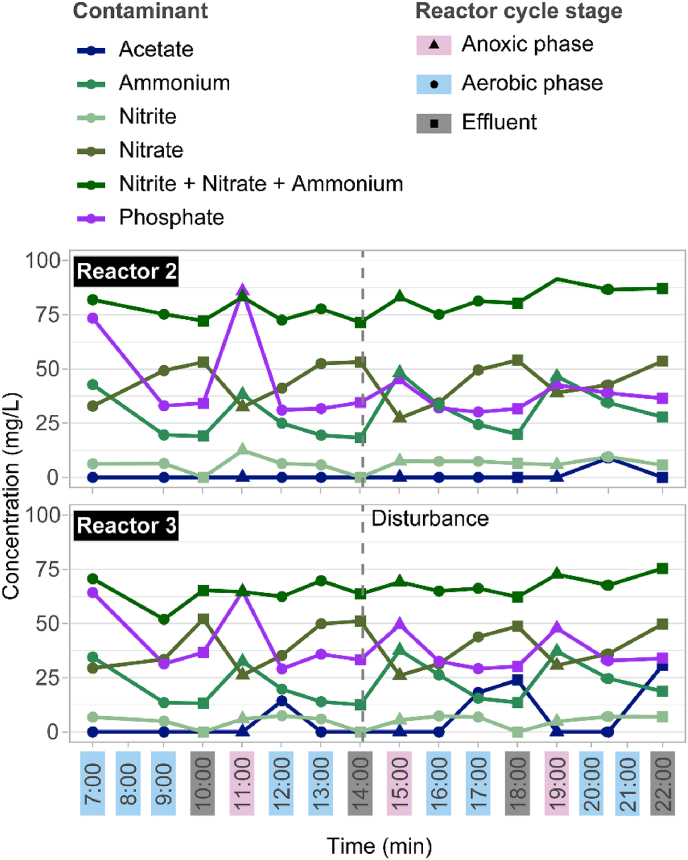
Cycle study results for reactors R2 and R3 during the third disturbance (day 100). The concentration of nutrients (ammonium, nitrite, nitrate, the sum of nitrate, nitrite and ammonium, and phosphate) are shown in the different reactor cycle stages. The concentration of nitrogen and phosphorous entering the reactors were 112.4 mL/g and 37.8 mL/g respectively. Vertical dashed lines indicate when the disturbance was applied to the reactors.

Overall, performance of R2 and R3 was very similar, showing similar trends during the experiment (Fig. 2E–J). TOC removal was stable during the experiment, always above 90%, with an average of 98% (SD = 0.8) for reactor R2 and 98% (SD = 0.3) for R3. An overall decreasing trend in total nitrogen (TN) concentration was observed, with an average removal of 35% (SD = 14.6) in reactor R2 and 37% (SD = 12.7) in reactor R3, peaking at day 64–86% in R2 and 70% in R3. The average ammonium removal was 86% (SD = 7.3) in R2 and 87% (SD = 7.1) in R3 but increased to 95% and 96% at the end of the experiment for R2 and R3 respectively. Denitrification occurred in the reactors as TN in the effluent was progressively reduced throughout the experiment, confirmed by the cycle studies showing nitrate depletion within the SBR cycles (Fig. 3). Phosphorus was removed in the reactors at an average of 31% (SD = 15.7) in R2 and 25% (SD = 12.5) in R3, with peaks of phosphorus removal in R2 at days 64 (71%) and 115 (79%) and in R3 at day 64 (66%) with significant differences between reactors during disturbance period II (Table S2). The results of the cycle study performed during the third disturbance did not show an important effect on nutrient removal as observed in Fig. 3. However, after the disturbance, the total nitrogen (the sum of ammonium, nitrite, nitrate) slightly increased, observed in the reactors as a slight reduction in ammonium and nitrite oxidation. Additionally, the anaerobic phosphorus release during the anaerobic phase, which accounted for a 60% and 40% phosphate concentration increment in R2 and R3 respectively, was lowered after the disturbance to 20% and 30% respectively because of the biomass removal.

### 3.2 The most abundant taxa had similar temporal trends in the two reactors

**Fig. 4:**
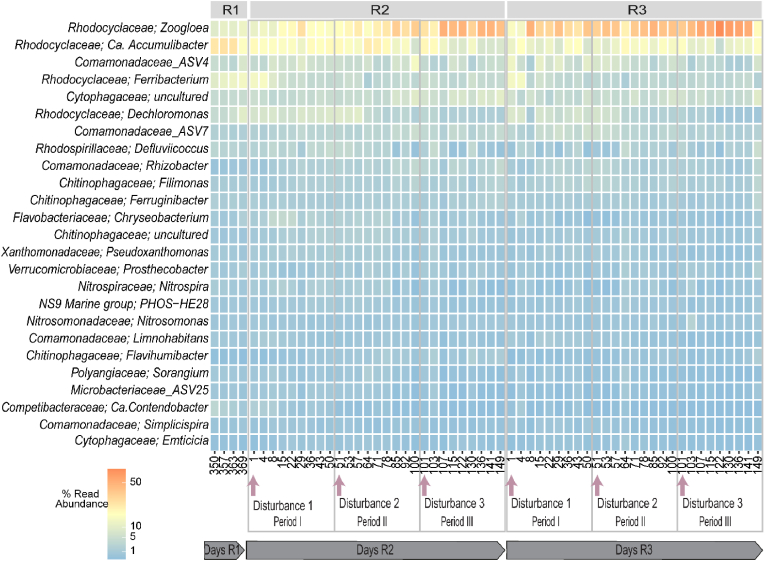
Temporal variation of the 25 most abundant genera as percentages of reads in the initial reactor (R1) and the replicate reactors (R2 and R3). The x-axis shows sampling days. The arrows and dashed lines indicate when the disturbances were applied to the reactor.

The temporal variation of the most abundant genera is displayed in Fig. 4 and relative abundances of ASVs are shown in Table S3. The microbial composition of the acclimatized inoculum sludge (R1) was dominated by the *Rhodocyclaceae* family (Fig. S3), with *Candidatus* Accumulibacter (hereafter entitled *Ca.* Accumulibacter), *Ferribacterium* sp., *Zoogloea* sp. and *Dechloromonas* sp. as the most abundant genera. During the disturbance stage, *Zoogloea* sp. (ASV1) and *Ca.* Accumulibacter (ASV2) were the dominant genera in the replicate reactors (Fig. 4). The applied disturbances caused *Zoogloea* sp. to show an increasing trend in relative abundance during the disturbance stage (Fig. S4), reaching percentages as high as 52% in R2 at day 107 and 73% in R3 at day 122 (Table S3). The relative abundance of *Ca.* Accumulibacter fluctuated during the experiment, with an overall decreasing trend (Fig. S4), ranging throughout the disturbance stage from 7% to 27% in R2 and from 4% to 22% in R3 (Table S3). Some bacteria such as *Ferribacterium* sp. and *Dechloromonas* sp. displayed a decreasing trend (Fig. S4), which was specially marked for *Ferribacterium* sp. (ASV3), part of the dominant community in the seed sludge, decreasing in abundance from 15% to ca. 4% and 2% in R2 and R3 respectively (Table S3). Other genera, such as *Rhizobacter* sp., *Flavihumibacter* sp. and ASV6 belonging to the family *Cytophagaceae*, were enriched during the experiment.

### 3.3 Alpha-diversity decreased over time

**Fig. 5:**
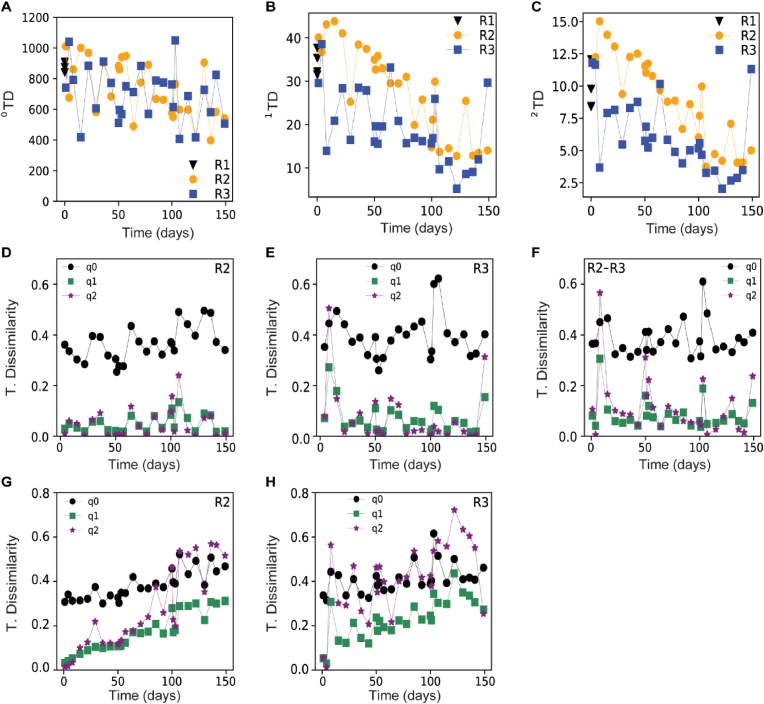
Dynamics over time in taxonomic (^q^TD) diversity. (A–C) alpha-diversity in reactor R1 (seed sludge), reactor R2 and reactor R3; (D–E) taxonomic beta-diversity between successive samples over time for R2 and R3, respectively; (F) taxonomic beta-diversity between reactors over time; (G–H) taxonomic beta-diversity between a given sample and the inoculum (R1, day 369) for R2 and R3, respectively. Disturbances were applied to the reactors the days 0, 50 and 100. The legend in D-H (q0, q1, q2) refers to diversity orders 0, 1, and 2.

A decreasing trend in alpha-diversity over time was observed for all diversity orders in both reactors. Both the taxonomic (^q^TD, Fig. 5A–C) and phylogenetic (^q^PD, Figs. S5A–C) alpha-diversity decreased over time with consistently higher diversity in R2 than R3 at the higher diversity orders. The periodic disturbances at day 0, 50 and 100 had unsystematic and limited effect on the alpha-diversity. In R3, alpha-diversity dropped by more than half after the first disturbance, while this was not seen in R2 (Fig. 5, S5). Furthermore, peaks in diversity were observed after the third disturbance in both reactors at all diversity orders, particularly obvious for ^q^PD in R3 (Fig. S5).

### 3.4 Disturbances led to a temporary increased in dissimilarity between the reactors

The microbial communities on the last day of the experiment were different from the seed sludge. Additionally, the communities in R2 and R3 were also different from each other (permanova, p = 0.001). This was true irrespective of the diversity order used to calculate community dissimilarity (q0, q1, and q2). However, analysis with the Raup-Crick null model suggested that the observed dissimilarity was smaller or equal to what could be expected from stochastic assembly. Each disturbance caused an increase in dissimilarity between the reactors, most clearly seen for higher diversity orders (Fig. 5F). This was especially marked for q1 and q2 after the first disturbance, increasing from values ca. 0.1 to 0.31 and 0.57 for the respective diversity orders. This dissimilarity returned to pre-disturbance levels within a few days, displaying an overall average of 0.4 for q0, 0.08 for q1, and 0.1 for q2 during the experiment. The taxonomic (Fig. 5D and E) and phylogenetic (Figs. S5D–E) dissimilarity between successive time points remained somewhat stable over time, with an overall average in taxonomic diversity of 0.4 for q0 in both R2 and R3, 0.06 and 0.07 for both q1 and q2, in R2 and R3 respectively. The dissimilarity between the seed sludge (R1) and the successive sample points increased with time (Fig. 5G–H, S5G-H), from 0.3 to 0.4 for both taxonomic and phylogenetic dissimilarities at diversity order q0 in both reactors. This was specially marked for higher diversity orders, increasing in both reactors from values close to 0 to 0.3 and ca. 0.5 for q1 and q2 respectively for the taxonomic dissimilarity and up to 0.7 and 0.6 for q1 and q2 respectively for the phylogenetic dissimilarity. The rate of change between pairwise samples over time, time-decay rates, for the different diversity orders was calculated for the whole experiment and the periods between each disturbance (Fig. S6). When the whole experiment was considered, the slope was significantly different from 0 for both reactors (p < 0.05) showing that the microbial community structure changed with time. When specific disturbance periods were considered separately, a significant community change was observed in R2 for Periods I and II for q1 and q2. Reactor R3 showed a high variability in the time-decay rate.

### 3.5 Granular sludge communities were stable with some changes in dominant taxa explained by stochasticity

**Fig. 6:**
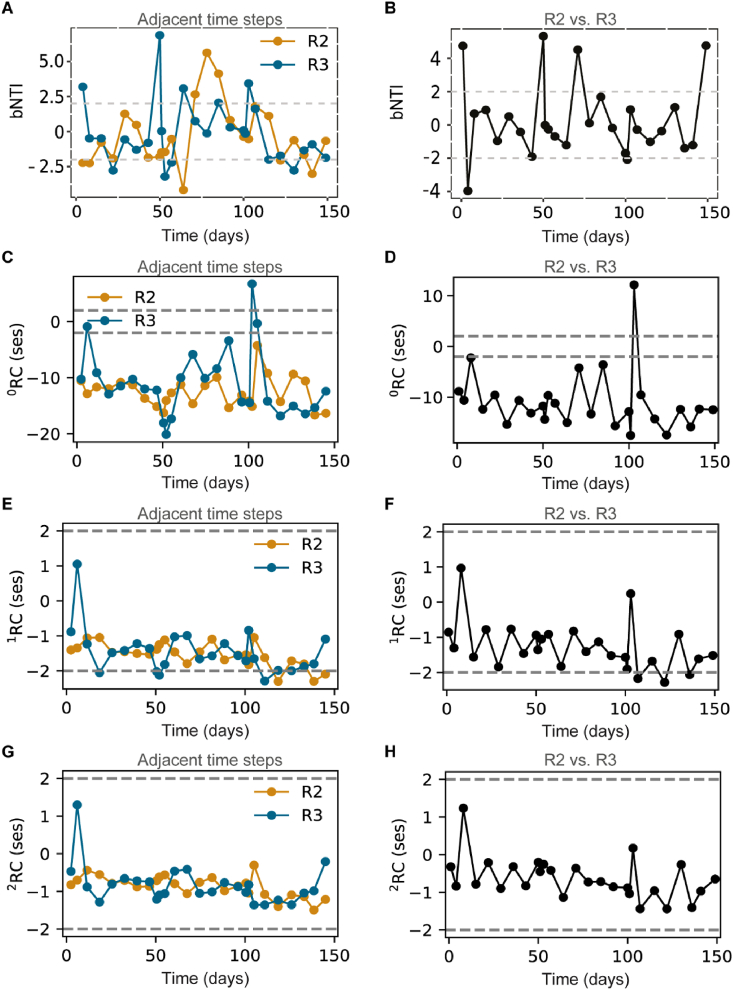
Null model analysis results for βNTI and ^q^RC. A, βNTI between two successive sample points; B, βNTI between replicate reactors; C, ^0^RC between two successive sample points; D, ^0^RC between the replicate reactors; E, ^1^RC between two successive sample points; F, ^1^RC between the replicate reactors; G, ^2^RC between two successive sample points; H, ^2^RC between the replicate reactors.

We used two different types of null models, βNTI (Fig. 6A and B), based on phylogenetic turnover, and ^q^RC (Fig. 6C–H), based on taxonomic turnover. Turnover between adjacent time points from the same reactor would show successional phases (Fig. 6A, C, E, G). Although fluctuating considerably over time, both the phylogenetic and taxonomic turnover were mostly similar to the null expectations, except ^0^RC, which was generally below −2. The same was observed for βNTI, and ^q^RC between reactors (Fig. 6B, D, E, F), which was generally not significantly different from the null expectations, except for ^0^RC.

## 4 Discussion

### 4.1 Community structure transition caused by periodical disturbances

In this study, the effect of the disturbance created by the removal of half of the reactor granular biomass was assessed by monitoring community diversity dynamics and reactor functions. For this, an acclimatized sludge to the reactor conditions was used, which was clearly dominated by polyphosphate-accumulating organisms (PAOs), such as *Ca.* Accumulibacter or *Dechloromonas* sp. The anaerobic pulse feeding strategy used in the reactors favoured bacteria that can accumulate storage polymers, like PAOs, which take up and store acetate as polyhydroxyalkanoate while degrading polyphosphate [62]. Two ASVs affiliated to *Ca.* Accumulibacter (ASV2) and *Dechloromonas* sp., (ASV5), clearly dominated the PAO community, with abundances reaching 30% and 9% of the total population respectively.

The disturbances applied here had two effects: a reduction of microbial biomass (ca. 50% and 30% average reduction in MLVSS and SRT respectively) and an increase in resource availability for the reactor microbiome (70% and 50% average increase in F/M ratio for R2 and R3, respectively). In granular reactors, the SRT vary between the microbial aggregates. Granules have long SRTs and slow-growing microorganism and habitat specialists are enriched, whereas slow-settling and smaller aggregates have short SRTs because they are withdrawn from the reactor due to the short settling time applied [12]. Thus, the removal of the biomass affected the selection for bigger microbial aggregates where PAOs and other slow-growing bacteria thrive. Additionally, the increase in F/M ratio after every disturbance increased the access to a readily available carbon substrate, which would favour r-strategist growing on acetate and replacing K-strategists [63,64]. Indeed, we observed some members of the abundant community in the acclimatized sludge to decrease during the disturbance period (i.e. *Ca.* Accumulibacter, *Ferribacterium* sp., *Dechloromonas* sp.) while others became enriched (i.e. *Zoogloea* sp., members of the *Comamonadaceae* and *Cytophagaceae* families). It is especially remarkable with the enrichment of ASV1 affiliated to *Zoogloea* sp. during the disturbance stage, accounting for ca. 13% in the inoculum and increasing to an average of ca. 26% and 30% of the communities in R2 and R3, respectively. The proliferation of *Zoogloea* sp. during the disturbance stage may be linked to the decrease of taxa that accumulate acetate during the anoxic phase, such as *Ca.* Accumulibacter-ASV2, which dominated the acclimatized sludge and was withdrawn from the reactors by the applied disturbances. This could have caused the leakage of acetate in the aerobic phase, favouring fast growing acetate consumers with a high substrate uptake rate like *Zoogloea* sp [65]. This was especially marked for reactor R3, with *Zoogloea* sp. reaching abundances as high as ca. 70% after the third perturbation when the SRT dropped from 18 days to 6 days. Additionally, we have previously observed *Zoogloea* sp. growing in the loosely packed layers around the granules, being more susceptible to erosion [66]. Thus, under these circumstances *Zoogloea* sp. could have also been enriched in the suspended phase growing on acetate.

Overall, the performance of the reactors was not significantly affected by disturbances despite the observed changes in the community structure. It has been previously reported that a stable performance of bioreactors treating wastewater is not necessarily concomitant to the stability of the microbial community [20,22]. This is due to the high degree of functional redundancy often found for general functions such as carbon oxidation. However, functions that are carried out by few taxa, such as nitrification and phosphorus removal, highly depend on the abundances of a narrow phylogenetically group of slow-growing microbes [67]. Indeed, we have previously observed the nitrifying community with abundances as low as 0.1% to be the main responsible of efficient nitrification in granular sludge reactors [68]. Therefore, the nitrifying community is especially sensitive to sludge loss and reduced SRT [65,68,69]. Certainly, here even if no significant changes were observed for nitrogen removal, a temporary increase in effluent ammonium concentration was observed following the disturbances. We also observed some differences for phosphorus concentration in the effluent between reactors, indicating a higher variability of this parameter, statistically significant during period III. The observed impact on nutrient removal could be linked to the removal of the sludge as these functional groups are enriched in large granules [70], and substrate competition caused by the leakage of acetate to the aerobic phase [71]. Even so, the dominant PAOs and the nitrifying community were kept in the reactors throughout the study period. The effects of the disturbances on the reactor performance did not show a clear long-term effect, the nutrient removal and granular integrity were maintained. These results are in agreement with those of [23]; which reported the maintenance of functional stability of aerobic granules despite changes in the composition of wastewater and disturbances caused by operation failures. These results highlight the stability and resilience of aerobic granular sludge systems under changing environment and disturbance regimes.

### 4.2 Resistance and resilience of microbial communities to disturbances

The diversity in the reactors was kept even when the disturbances affected the community structure, as alpha-diversity did not experience a marked drop after each disturbance in any of the reactors, but rather a steady reduction. Therefore, we could infer that the communities were somewhat resistant to the disturbances. Additionally, the results support the resilience of the microbial community towards the applied disturbances. The dissimilarity between reactors was rather stable between disturbances, only increasing after each disturbance and then rapidly recovering to the previous state, evidencing the importance of deterministic factors (i.e., reactor conditions) during community assembly. It is, however, difficult to disentangle differences in diversity due to successional patterns in the microbial community structure from changes in diversity due to the disturbances applied to the reactor. Here, the community in both reactors experienced a decreasing trend in alpha-diversity and an increasing trend in dissimilarity between the seed sludge and the successive sample points. Decay rates indicated slow but significant change in community structure over the entire experimental period. This is consistent with other bioreactor studies [40,72] where the microbial communities adapted to those conditions are selected and biodiversity is reduced as a result of both extinction and competitive exclusion. It is, however, important to bear in mind that the inoculum sludge was acclimatized for 369 days before it was used to inoculate reactors R2 and R3. The only parameter that was changed was the settling time, from 30 to 2 min, and even though the sludge was already granulated, it is possible that this influenced the microbial community by the wash-out of smaller microbial aggregates. The dominant fraction of the community also displayed higher dynamics in both alpha- and beta-diversity and an increasing trend in dissimilarity between the seed and successive sample points, especially for the phylogenetical beta-diversity. This is consistent with the reduction of the dominant slow-growing ASVs growing in large granules, which were withdrawn from the system at each disturbance. Interestingly, R2 seemed to be somewhat more resistant to the disturbances than R3, as it displayed a lower diversity loss at higher diversity orders, a lower dispersion in the time-decay rate and a more stable successional trend. This might be due to the sharp decrease in alpha-diversity experienced in R3 after the first disturbance, making the community more susceptible to further disturbances [73].

The taxonomic and phylogenetic turnover over time and between reactors suggested that the changes of the abundant members of the community (measured at higher orders of q) were similar to the null expectation. This indicates that stochastic processes were the major ecological mechanism responsible for the community succession. We have previously observed drift to become relevant once the microbial communities reach successional stability under constant reactor conditions [40]. The long acclimation period prior to the operation of R2 and R3 may explain why mainly stochastic factors were shaping the communities. The disturbances did not change the importance of stochasticity, nor did exert a strong selective pressure on the community. This apparent contradiction with the observed changes in the community structure resulting from the applied disturbances could be explained by the random picking of granules when the sludge was removed from the reactors, as community variability within granules is large [74]. The applied disturbances were likely of too low intensity to have caused deterministic factors to become more relevant than those stochastic for the overall community assembly. This dominance of internal community ecological processes over external selective pressures has been reported before [75,76]. [77] showed in reactors with activated sludge that stochastic assembly was relatively most important under intermediate-level disturbances, with deterministic forces growing in importance at high-level disturbances. Here, the stochastic particle purge, together with the prior dominance of drift in community assembly would explain the stochastic community turnover.

## 5 Conclusions

Here, the effect of disturbances in two replicate reactors were studied for 149 days. The removal of large granules where slow growing bacteria were enriched, had a slight effect on specialized functions; yet the overall reactor performance was not importantly affected. Using null models together with taxonomic and phylogenetic diversity analysis we show that, overall, the microbial communities were resistant to the disturbances. The observed differences in community structure between reactors and in the succession were mainly driven by changes in alpha-diversity and not turnover. Some turnover occurred however, with stochasticity being the main driving force for these community changes, with community drift and random granule purge governing the overall community assemblage of the acclimatized sludge. The results altogether suggest that the community showed resistance towards the applied disturbances, evidencing the aerobic granular sludge process as a robust system for wastewater treatment.

## CRediT authorship contribution statement

**Raquel Liébana:** Conceptualization, Data curation, Formal analysis, Investigation, Methodology, Visualization, Writing – original draft, Writing – review & editing. **Oskar Modin:** Conceptualization, Data curation, Formal analysis, Funding acquisition, Methodology, Supervision, Visualization, Writing – review & editing. **Frank Persson:** Conceptualization, Funding acquisition, Methodology, Supervision, Formal analysis, Writing – review & editing. **Malte Hermansson:** Conceptualization, Funding acquisition, Writing – review & editing. **Britt-Marie Wilén:** Conceptualization, Formal analysis, Funding acquisition, Methodology, Project administration, Supervision, Writing – review & editing.

## Declaration of competing interest

The authors declare that they have no known competing financial interests or personal relationships that could have appeared to influence the work reported in this paper.

## Data Availability

Data will be made available on request.
