# Supplementary material for: Resistance of aerobic granular sludge microbiomes to periodic loss of biomass
Source: Biofilm. 2023 Jul 28;6:100145. doi: 10.1016/j.bioflm.2023.100145 (PMC10415711; doi:10.1016/j.bioflm.2023.100145)
Supplement: Multimedia component 1 [file mmc1.docx]

**Supplementary material**

| Day | p |
| --- | --- |
| 8 | 0.08 |
| 22 | 0.71 |
| 50 | 0.24 |
| 57 | 0.59 |
| 78 | 0.22 |
| 92 | 0.80 |
| 112 | 0.24 |
| 130 | 0.59 |
| 142 | 0.22 |

**Table S1.** Results of paired sample Wilcoxon signed-rank tests performed on granule size in the reactors for each sampling date. P-values (*<0.05) are provided.

**Table S2.** Results of paired sample Wilcoxon signed-rank tests comparing R2 and R3 in periods I-III (P) for the parameters total suspended solids in the reactor (MLSS), volatile suspended solids in the reactor (MLVSS), total suspended solids in the effluent (EffTSS), volatile suspended solids in the effluent (EffVSS), sludge retention time (SRT) and effluent concentrations of total organic carbon concentrations (TOC), Acetate, total nitrogen (TN), ammonium (NH_4_^+^), nitrite (NO_2_^-^), nitrate (NO_3_^-^), the sum of ammonium, nitrite and nitrate (NO_2_^-^-NO_3_-NH_4_^+^) and phosphate (PO_4_^3-^). P-values (*<0.05) are provided.

| P | MLSS | MLVSS | EffTSS | EffVSS | SRT | TOC | Acetate | TN | NH_4_^+^ | NO_3_^-^ | NO_2_^-^ | NO_2_^-^-NO_3_-NH_4_^+^ | PO_4_^3-^ |
| --- | --- | --- | --- | --- | --- | --- | --- | --- | --- | --- | --- | --- | --- |
| 1 | 0.35 | 0.48 | 0.25 | 0.03* | 0.38 | 0.55 | 1.00 | 0.84 | 1 | 0.26 | 0.10 | 0.22 | 0.15 |
| 2 | 0.22 | 0.26 | 0.61 | 0.62 | 0.62 | 0.17 | 0.40 | 0.62 | 0.66 | 0.15 | 1.00 | 0.82 | 0.04* |
| 3 | 0.58 | 0.56 | 0.15 | 0.01* | 0.44 | 0.33 | 0.60 | 0.53 | 0.25 | 0.73 | 0.37 | 0.73 | 0.11 |

**Table S3**. Relative abundance of ASVs retrieved during the microbial succession in reactors R1, R2 and R3.

*Excel dataset Table S3*


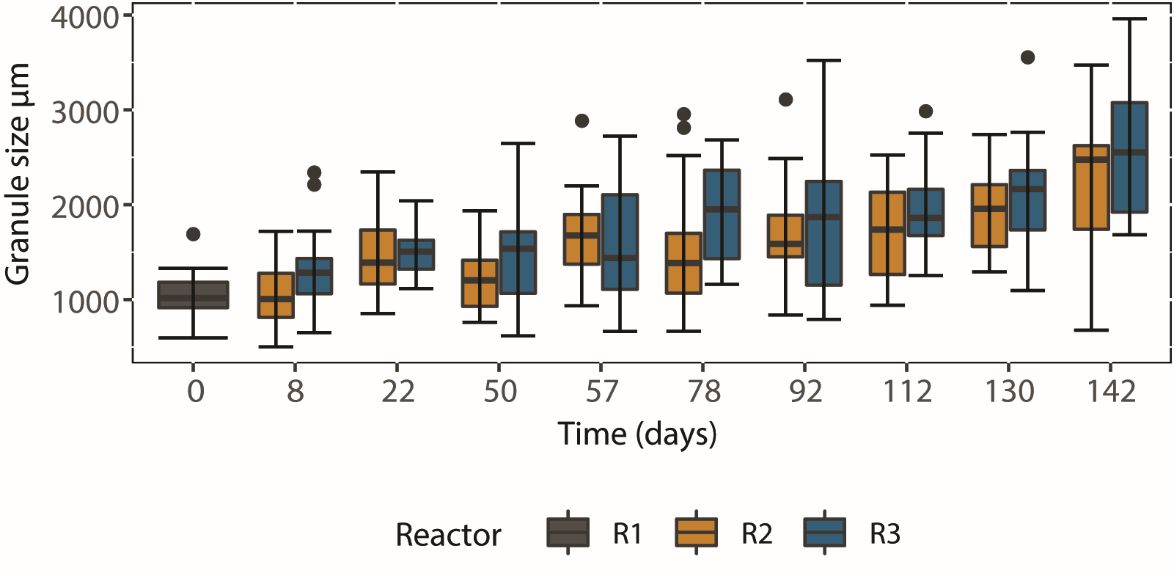


**Figure S1.** Particle size of the seed (R1) and the replicate reactors R2 and R3 during the experiment (n=15).


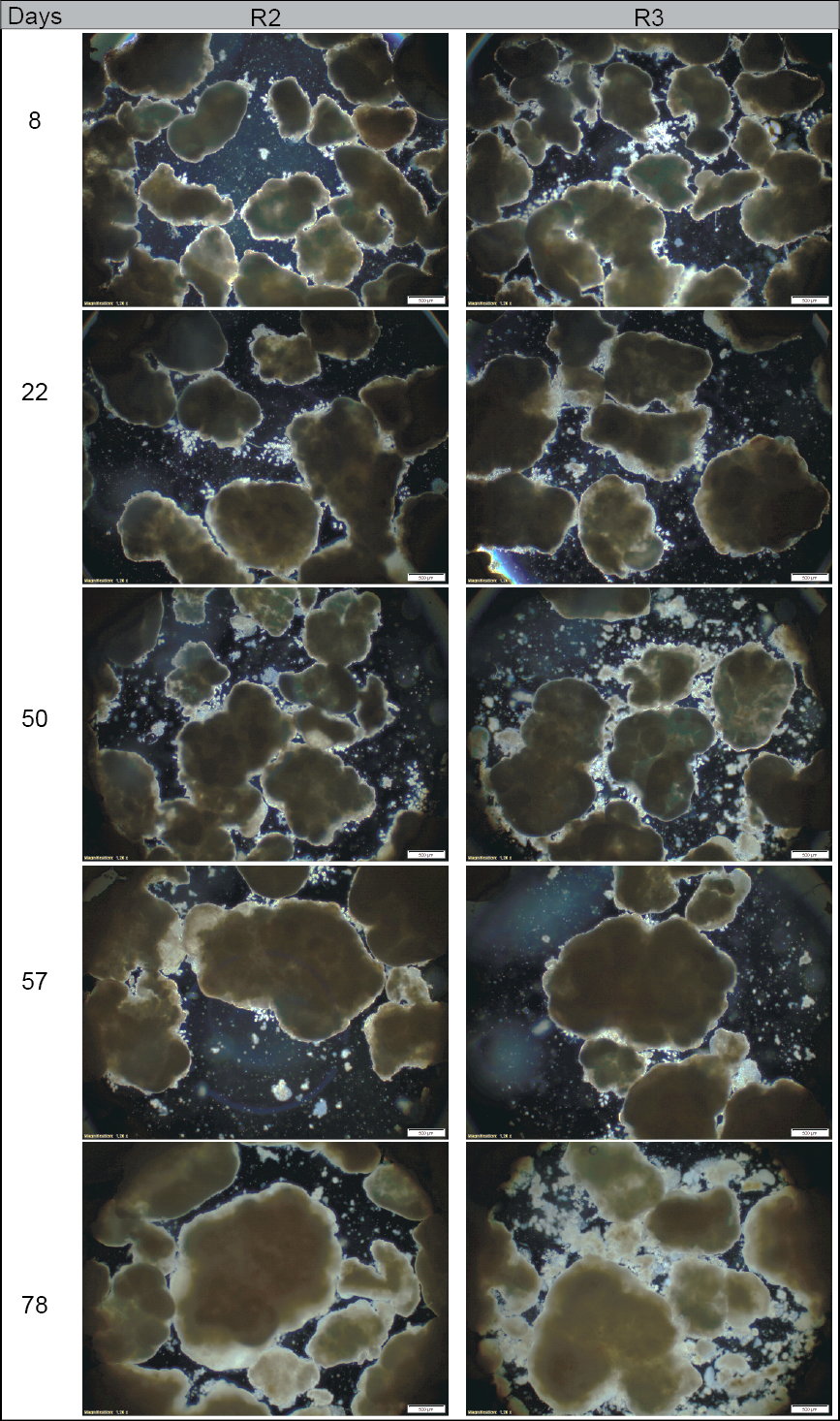


*Cont.*


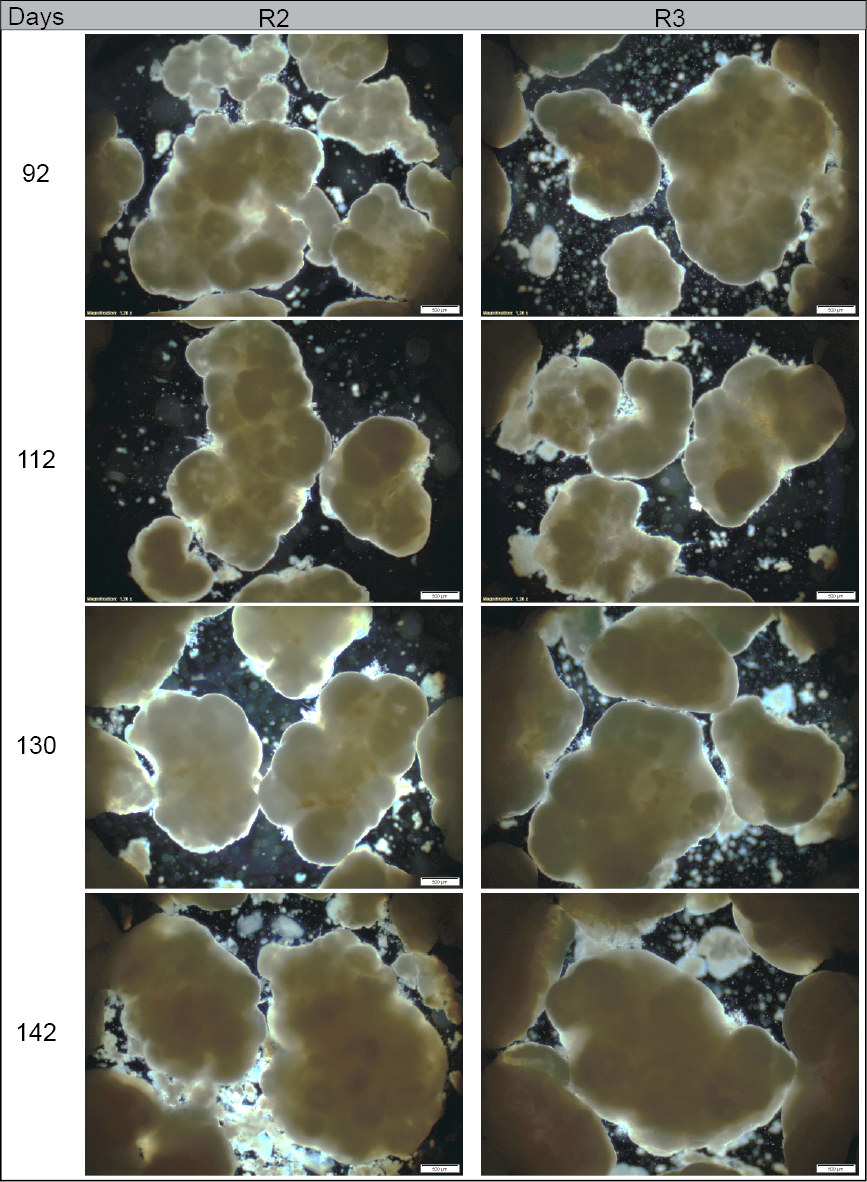


**Figure S2.** Microscopic observations of the granular sludge over time in reactors R2 and R3. The scale bars designate 500 µm.


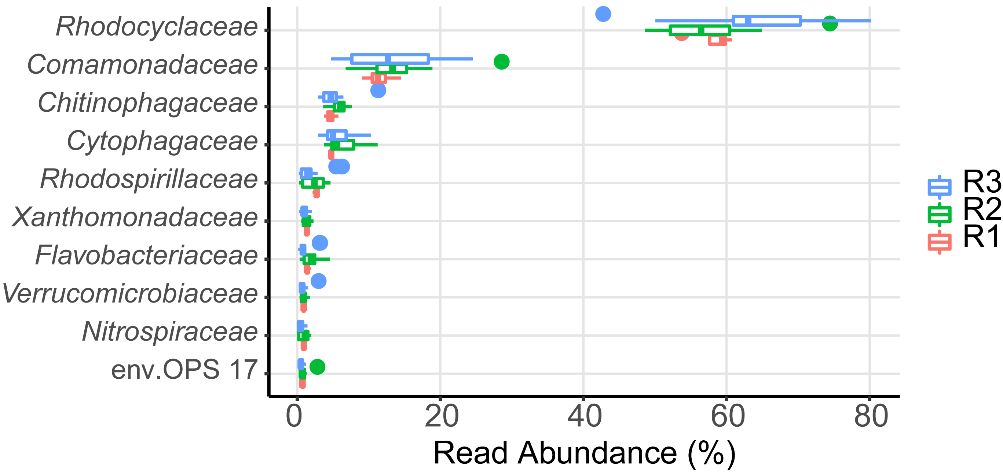


### **Figure S3.** Box plots showing the relative read abundances of the 10 most abundant families in the replicate reactors (R1, R2 and R3).


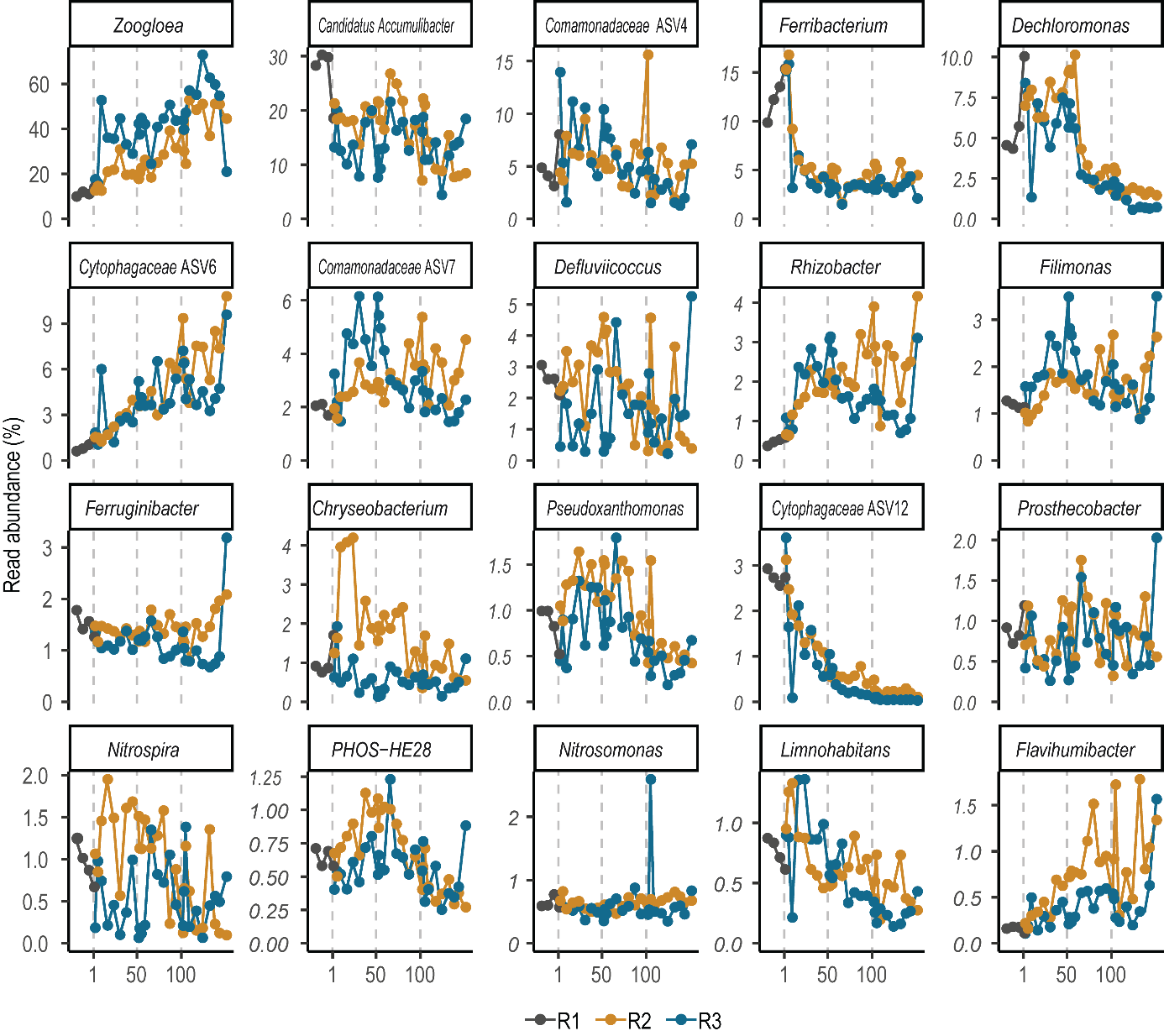


### **Figure S4.** Time-series plot showing the relative read abundances of the 20 most abundant genera in the replicate reactors (R1, R2 and R3).


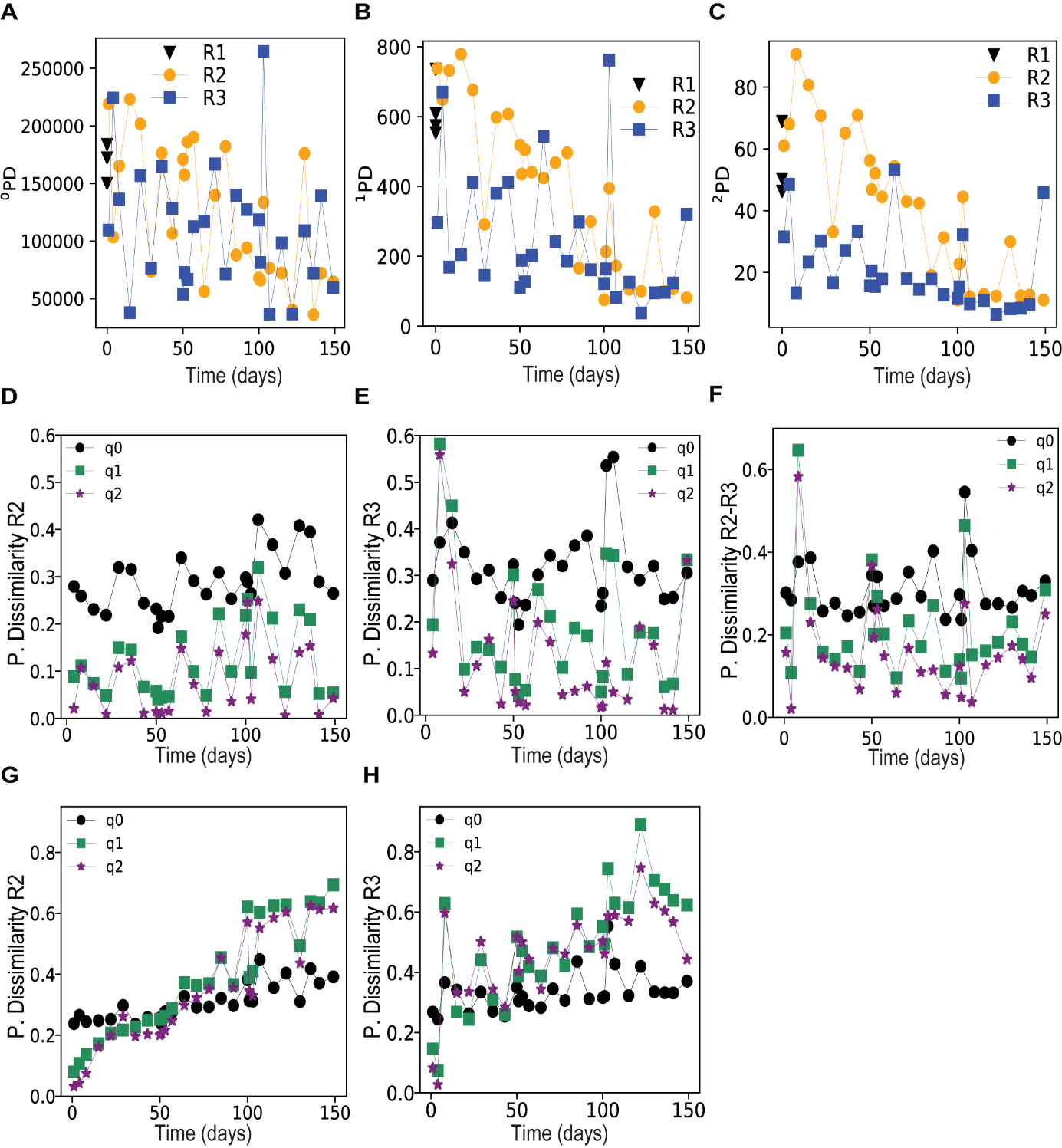


**Figure S5.** Dynamics of the phylogenetic (PD) alpha-diversity (A-C) during the experiment in reactor R1 (seed sludge), reactor R2 and reactor R3; phylogenetic beta-diversity between successive samples over time for R2 (D) and R3 (E) respectively; phylogenetic beta-diversity between reactors over time (F); phylogenetic beta-diversity between a given sample and the inoculum (R1, day 369) for R2 (G) and R3 (H) respectively. At a q of 0, all ASVs are considered equally important; at a q of 1, ASVs are weighted according to the relative abundance; at a q of 2, abundant ASVs are given a larger weight. The β-diversities were converted into dissimilarity indices constrained between 0 (two identical samples) and 1 (two samples with no shared ASVs). Disturbances were applied to the reactors on days 0, 50 and 100.


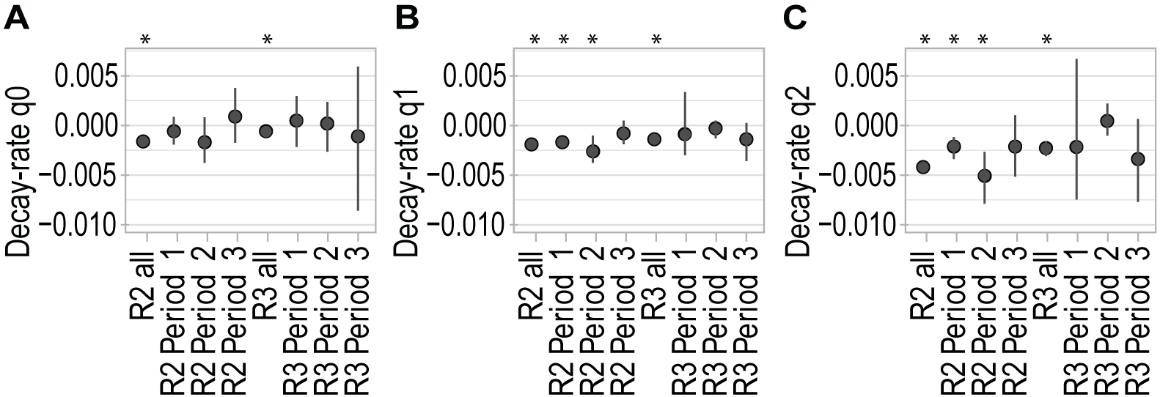


**Figure S6.** Time-decay rates in reactors R2 and R3 calculated for the whole experiment and the periods between each disturbance for Hill numbers q0 diversity order (A), for Hill numbers q1 diversity order (B) and for Hill numbers q2 diversity order (C). Asterisk (*) indicate time-decay rates being significantly different from zero (p<0.05, based on bootstrapping and 999 randomizations).
